# Parada Cardiorrespiratória Extra-Hospitalar durante a Pandemia da Doença por Coronavírus 2019 (COVID-19) no Brasil: A Mortalidade Oculta

**DOI:** 10.36660/abc.20210041

**Published:** 2021-02-19

**Authors:** Claudio Tinoco Mesquita

**Affiliations:** 1 Universidade Federal FluminenseNiteróiRJBrasilUniversidade Federal Fluminense, Niterói, RJ - Brasil; 2 Hospital Pró-CardíacoRio de JaneiroRJBrasilHospital Pró-Cardíaco, Rio de Janeiro, RJ - Brasil; 3 Hospital VitóriaRio de JaneiroRJBrasilHospital Vitória, Rio de Janeiro, RJ – Brasil

**Keywords:** COVID-19/complicações, Betacoronavírus, Mortalidade, Pandemia, Parada Cardíaca, Fatores de Risco, Idoso, Doenças Cardiovasculares, Pneumopatias

## Abstract

O mundo mudou em apenas alguns meses após o surgimento da doença do novo coronavírus 2019 (COVID-19), causada por um betacoronavírus denominado síndrome respiratória aguda grave por coronavírus 2 (SARS-CoV-2). A COVID-19 foi declarada uma pandemia pela Organização Mundial da Saúde (OMS) em 11 de março de 2020. O Brasil apresenta atualmente o segundo maior índice de mortalidade por COVID-19 do mundo, perdendo apenas para os EUA. A pandemia da COVID-19 está se espalhando rapidamente pelo mundo, com mais de 181 países afetados. O presente editorial se refere ao artigo publicado nos Arquivos Brasileiros de Cardiologia: “Aumento de óbitos domiciliares devido a parada cardiorrespiratória em tempos de pandemia de COVID-19”^1^ Seus principais resultados mostram um aumento gradual na taxa de paradas cardiorrespiratórias extra-hospitalares durante a pandemia da doença por coronavírus 2019 (COVID-19) na cidade de Belo Horizonte, Minas Gerais, Brasil. Seus dados demonstram um aumento proporcional de 33% dos óbitos domiciliares em março de 2020 em relação aos períodos anteriores. O estudo é o primeiro artigo brasileiro a demonstrar a mesma tendência observada em outros países.

“Em algum lugar, algo incrível está esperando para ser descoberto”.

**Carl Sagan**

Meu interesse pessoal pela Ciência deve ser creditado a Carl Sagan. Durante minha juventude, tive a oportunidade de assistir seu programa de TV “Cosmos” e isso mudou tudo. Hoje, a Ciência é uma das principais prioridades da humanidade. O mundo mudou em apenas alguns meses após o surgimento da doença do novo coronavírus 2019 (COVID-19), causada por um betacoronavírus denominado síndrome respiratória aguda grave por coronavírus 2 (SARS-CoV-2). A COVID-19 foi declarada uma pandemia pela Organização Mundial da Saúde (OMS) em 11 de março de 2020.^2^ O Brasil apresenta atualmente o segundo maior índice de mortalidade por COVID-19 do mundo, perdendo apenas para os EUA. A pandemia de COVID-19 está se espalhando rapidamente pelo mundo, com mais de 181 países afetados.

Na maioria dos pacientes, a COVID-19 é uma doença leve com alguns sintomas respiratórios. A COVID-19 é mais grave e fatal entre pacientes com fatores de risco cardiovascular ou doenças preexistentes.^3^ O Centro Chinês para Controle e Prevenção de Doenças publicou uma pesquisa que demonstrou que entre os pacientes com diagnóstico de COVID-19, 13% tinham hipertensão, 5% tinham diabetes mellitus e 4% tinham histórico de doença cardiovascular. Porém, na mesma coorte, entre os pacientes que não sobreviveram, 40% eram hipertensos, 20% eram diabéticos e 22% apresentavam doenças cardiovasculares preexistentes.^4^ Pacientes com doenças cardiovasculares apresentaram a maior taxa de letalidade (10,5%). Os fatores de risco de eventos cardíacos após a pneumonia por COVID-19 incluem idade avançada, doenças cardiovasculares pré-existentes e maior gravidade da pneumonia na apresentação.^2^ Doenças coronarianas também estão associadas a eventos cardíacos agudos e desfechos desfavoráveis na influenza e outras infecções virais respiratórias.^5,6^ A COVID-19 também demonstrou danos ao sistema cardiovascular com manifestações diversas como lesão miocárdica, infarto agudo do miocárdio, insuficiência cardíaca, síndrome de Takotsubo (ST), arritmias, miocardite e choque.^7^ Não apenas as condições cardiovasculares crônicas como hipertensão ou insuficiência cardíaca são relevantes para os desfechos da COVID-19, mas também a idade, o estado imunológico do hospedeiro e o efeito de medicamentos para doenças cardiovasculares como antitrombóticos ou anti-hipertensivos.^8^ A Figura 1 demonstra a interação entre doenças cardiovasculares/fatores de risco e COVID-19.

**Figura 1:**
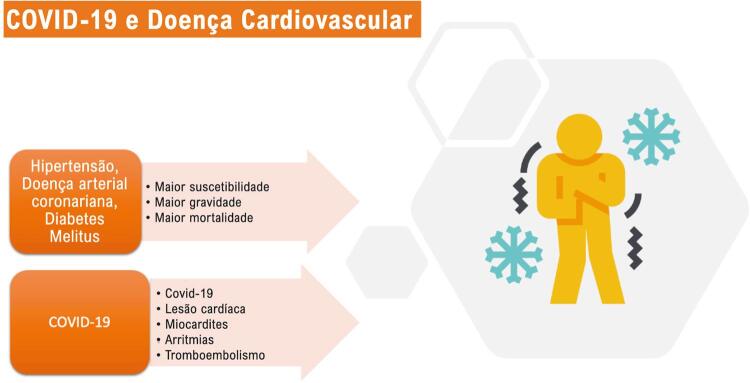
– Interação entre doenças cardiovasculares/fatores de risco e COVID-19. Comorbidades cardiovasculares como hipertensão e doença arterial coronariana estão associadas a maior suscetibilidade e maior mortalidade em pacientes com COVID-19. A COVID-19 também está associada a manifestações cardiovasculares, incluindo lesão miocárdica, miocardite, arritmias, síndrome coronariana aguda e tromboembolismo.

Os danos que a COVID-19 causa no sistema cardiovascular provavelmente são multifatoriais e podem resultar de um desequilíbrio entre alta demanda metabólica e baixa reserva cardíaca, inflamação sistêmica e trombogênese, além de dano cardíaco direto provocado pelo vírus.^7^ As complicações do sistema cardiovascular ocorrem principalmente em pacientes com fatores de risco cardiovascular (idade avançada, hipertensão e diabetes) ou doenças cardiovasculares preexistentes. Existem poucos relatos de pacientes com COVID-19 que apresentaram supradesnivelamento agudo do segmento ST e, para esses pacientes, as alterações ECG estavam presentes nas derivações inferiores. Em cada caso, o diagnóstico de miocardite foi apoiado por troponinas cardíacas elevadas, diminuição moderada da fração de ejeção do ventrículo esquerdo e ausência de doença arterial coronariana com limitação de fluxo por angiocoronariografia invasiva.^9^ Os achados da autópsia corroboram o conceito de que a patogênese da COVID-19 grave envolve lesão direta induzida por vírus em múltiplos órgãos, incluindo coração e pulmões, juntamente com as consequências de um estado pró-coagulante com coagulopatia.^10^ A Tabela 1 lista as consequências cardiovasculares mais frequentes da COVID-19 descritas na literatura.

**Tabela 1:** – Consequências cardiovasculares da COVID-19

Complicação Cardiovascular	Frequência	Implicações
Lesão miocárdica e miocardite	7–20% dos pacientes com COVID-19	A lesão miocárdica está associada a um aumento de 5 vezes na necessidade de ventilação mecânica invasiva e a um aumento de 11 vezes na mortalidade
Síndrome coronariana aguda (SCA)	Menos de 5% dos pacientes com COVID-19	Redução nas hospitalizações por SCA e redução de 40% no infarto do miocárdio com supradesnivelamento do segmento ST durante a pandemia. Essa redução nos casos de SCA está associada a um aumento semelhante nas paradas cardiorrespiratórias extra-hospitalares.
Insuficiência cardíaca	Incidência de 24% em todos os pacientes com COVID-19 e 49% em pacientes que vieram a óbito	A insuficiência cardíaca pode contribuir para a insuficiência respiratória em pacientes com SDRA. O manejo adequado da IC é obrigatório para reduzir a mortalidade.
Arritmias e parada cardiorrespiratória súbita	Arritmias malignas como taquicardia ventricular e fibrilação podem ocorrer em pacientes com níveis elevados de troponina T	Taquicardia atrial e ventricular e fibrilação podem ser desencadeadas por lesão miocárdica, causas sistêmicas ou interações medicamentosas. É necessária atenção especial ao prolongamento do intervalo QT induzido por drogas
Alterações de coagulação e trombose	Níveis elevados de dímero-d (>0,5 mg/l) podem ser encontrados em 60% dos indivíduos com doenças graves	Não se tem conhecimento do regime ideal de anticoagulação para prevenir eventos tromboembólicos, mas, geralmente, a anticoagulação é prescrita empiricamente. Recomendações sobre tratamento antitrombótico e ICP para síndromes coronarianas agudas devem ser mantidas durante o tratamento da COVID-19

SDRA: síndrome do desconforto respiratório agudo; ICP: intervenção coronariana percutânea (adaptado de Nishiga et al.^11^ Prieto-Lobato et al.^12^

Em relação à epidemiologia da doença arterial coronariana, alguns estudos mostraram diminuição na incidência de hospitalização por infarto agudo do miocárdio durante a pandemia de Covid-19. Solomon et al.,^13^ observaram que as taxas semanais de hospitalização por infarto agudo do miocárdio diminuíram em até 48% durante o período da COVID-19(12). De Filippo et al.,^14^ encontraram diminuição semelhante na hospitalização por síndrome coronariana aguda em 15 hospitais no norte da Itália.^14^ Essa diminuição está parcialmente relacionada à ansiedade e ao medo que muitos pacientes demonstraram durante os meses iniciais da pandemia de contrair a COVID-19 nas unidades de emergência. Devido a essa diminuição nas internações por infarto do miocárdio, observou-se um aumento transitório de paradas cardiorrespiratórias extra-hospitalares (PCEH) em comparação ao mesmo período nos anos anteriores à pandemia.^15^ Esse aumento de casos de PCEH é diretamente atribuível às infecções por COVID-19 e ao possível aumento de pacientes com síndrome coronariana aguda que não se dirigiram imediatamente aos serviços de emergência.

Devemos parabenizar os autores do artigo publicado nos Arquivos Brasileiros de Cardiologia.^1^ Seus principais achados mostram um aumento gradual na taxa de paradas cardiorrespiratórias extra-hospitalares durante a pandemia de COVID-19 na cidade de Belo Horizonte, Minas Gerais, Brasil. Seus dados demonstram um aumento proporcional de 33% nos óbitos domiciliares em março de 2020 em relação aos períodos anteriores. O estudo é o primeiro artigo brasileiro a demonstrar a mesma tendência observada em outros países.^14,15^ As principais limitações do estudo são: curto período de observação, amostra de uma única região metropolitana brasileira e a falta de informações completas sobre comorbidades em cerca de 40% dos casos. No entanto, esses dados não invalidam as principais mensagens do estudo, que são: (1) a necessidade de organizar o sistema de saúde para lidar com os casos de doenças agudas durante a pandemia de COVID-19, (2) conscientizar a população sobre a necessidade de buscar cuidados continuados de saúde e (3) a busca por melhores tratamentos e prevenção.

A aprovação de vacinas eficazes para a prevenção da COVID-19 e o início do programa nacional de imunização contra a COVID-19 em janeiro de 2021 nos enchem de esperança e otimismo.^16,17^ Porém, enquanto a vacinação não estiver amplamente disponível para a população, é necessário dar continuidade a medidas eficazes e cientificamente comprovadas de distanciamento social, uso de máscaras e higienização das mãos. Só então a pandemia de COVID-19 será uma página da história e não mais uma dura realidade.
