# Supplementary material for: Parada Cardiorrespiratória Extra-Hospitalar durante a Pandemia da Doença por Coronavírus 2019 (COVID-19) no Brasil: A Mortalidade Oculta
Source: Arq Bras Cardiol. 2021 Feb 19;116(2):272–4. [Article in Portuguese] doi: 10.36660/abc.20210041 (PMC7909974; doi:10.36660/abc.20210041)
Supplement: Translation

# Translation: Out-Of-Hospital Cardiac Arrest during the Coronavirus Disease 2019 (COVID-19) Pandemic in Brazil: The Hidden Mortality

**Authors:** Claudio Tinoco Mesquita

**Affiliations:** 1 BrasilUniversidade Federal Fluminense, Niterói, RJ - Brasil; 2 BrasilHospital Pró-Cardíaco, Rio de Janeiro, RJ - Brasil; 3 BrasilHospital Vitória, Rio de Janeiro, RJ – Brasil

**Keywords:** COVID-19/complications, Betacoronavirus, Mortality, Pandemics, Heart Arrest, Risk Factors, Elderly, Cardiovascular Diseases, Pneumopathies

## Abstract

The world changed in just a few months after the emergence of the novel coronavirus disease 2019 (COVID-19), caused by a beta coronavirus named severe acute respiratory syndrome coronavirus 2 (SARS-CoV-2). COVID-19 was declared a pandemic by the World Health Organization (WHO) on March 11, 2020. Brazil currently has the world’s second-highest COVID-19 death toll, second only to the USA. The COVID-19 pandemic is spreading fast in the world with more than 181 countries affected. This editorial refers to the article published in Arquivos Brasileiros de Cardiologia: “Increase in home deaths due to cardiorespiratory arrest in times of COVID-19 pandemic.”^1^ Their main results show a gradual increase in the rate of out-of-hospital cardiac arrest during the Coronavirus disease 2019 (COVID-19) pandemic in the city of Belo Horizonte, Minas Gerais, Brazil. Their data demonstrate a proportional increase of 33% of home deaths in March 2020 compared to previous periods. Their study is the first Brazilian paper to demonstrate the same trend observed in other countries.

“Somewhere, something incredible is waiting to be known.”

**Carl Sagan**

My personal interest in Science must be credited to Carl Sagan. During my youth, I saw his TV program “Cosmos” and this changed everything. Today, Science is one of the top priorities of humankind. The world changed in just a few months after the emergence of the novel coronavirus disease 2019 (COVID-19), caused by a beta coronavirus named severe acute respiratory syndrome coronavirus 2 (SARS-CoV-2). COVID-19 was declared a pandemic by the World Health Organization (WHO) on March 11, 2020.^2^ Brazil currently has the world’s second highest Covid-19 death toll, second only to the USA. The COVID-19 pandemic is spreading fast in world with more than 181 countries affected.

In the majority of patients, COVID-19 is a mild disease with some respiratory symptoms. COVID-19 is more severe and fatal among patients with pre-existing cardiovascular risk factors or diseases.^3^ The Chinese Center for Disease Control and Prevention published a survey demonstrating that among patients diagnosed with COVID-19, 13% had hypertension, 5% had diabetes mellitus and 4% had a history of cardiovascular disease. However, in the same cohort, among patients who had not survived, 40% had hypertension, 20% had diabetes and 22% had pre-existing cardiovascular diseases.^4^ Patients with cardiovascular diseases had the highest case fatality rate (10.5%). Risk factors of cardiac events after COVID-19 pandemic include older age, pre-existing cardiovascular diseases and greater severity of pneumonia at presentation.^2^ Coronary heart disease has also been found to be associated with acute cardiac events and poor outcomes in influenza and other respiratory viral infections.^5,6^ COVID-19 has also demonstrated damage to the cardiovascular system with several manifestations, such as myocardial injury, acute myocardial infarction, heart failure, Takotsubo syndrome (TS), arrhythmias, myocarditis and shock.^7^ Not only chronic cardiovascular conditions, such as hypertension or heart failure, are relevant to the COVID-19 outcomes, but also age, immunological status of the host and the effect of cardiovascular drugs like antithrombotic drugs or antihypertensives.^8^Figure 1 demonstrates the interaction between cardiovascular diseases/risk factors and COVID-19.

**Figure 1:** – Interaction between cardiovascular diseases/risk factors and COVID-19. Cardiovascular comorbidities such as hypertension and coronary artery disease are associated with increased susceptibility and higher mortality in patients with COVID-19. COVID-19 is also associated with cardiovascular manifestations including myocardial injury, myocarditis, arrhythmias, acute coronary syndrome and thromboembolism.

The damage that COVID-19 causes in the cardiovascular system is probably multifactorial and can result from an imbalance between high metabolic demand and low cardiac reserve, systemic inflammation and thrombogenesis, in addition to direct cardiac damage from the virus.^7^ Cardiovascular system complications occur mainly in patients with cardiovascular risk factors (advanced age, hypertension and diabetes) or preexisting cardiovascular diseases. There are few reports of COVID-19 patients who presented with acute ST-segment elevations and, for those patients, the ECG changes were present in the inferior leads. In each case, a diagnosis of myocarditis was supported by elevated cardiac troponins, a moderate decrease of left ventricular ejection fraction and the absence of flow-limiting coronary artery disease by invasive coronary angiography.^9^ Autopsy findings support the concept that the pathogenesis of severe COVID-19 disease involves direct viral-induced injury of multiple organs, including heart and lungs, coupled with the consequences of a procoagulant state with coagulopathy.^10^Table 1 lists the most frequent cardiovascular consequences of COVID-19 described in the literature.

**Table 1:** – Cardiovascular consequences of COVID-19

Cardiovascular Complication	Frequency	Implications
Myocardial injury and myocarditis	7–20% of patients with COVID-19	Myocardial injury is associated with a 5-fold increase in the need for invasive mechanical ventilation and an 11-fold increase in mortality
Acute coronary syndrome (ACS)	Less than 5% of patients with COVID-19	Reduction in hospitalizations for ACS and a 40% reduction in ST-segment elevation myocardial infarction during the pandemic. This decrease in ACS cases is associated with a similar increase in out-of-hospital cardiac arrest.
Heart failure	Incidence of 24% in all patients with COVID-19 and 49% in patients who died	Heart failure can contribute to respiratory failure in patients with ARDS. Adequate management of HF is mandatory to reduce mortality.
Arrhythmias and sudden cardiac arrest	Malignant arrhythmias, such as ventricular tachycardia and fibrillation, can occur in patients with elevated levels of troponin T	Atrial and ventricular tachycardia and fibrillation can be triggered by myocardial injury, systemic causes or drug interactions. Special attention is needed with drug-induced QT prolongation
Coagulation abnormalities and thrombosis	Elevated levels of d-dimer (>0.5 mg/l) can be found in 60% of those with severe illnesses	Optimal anticoagulation regimen to prevent thromboembolic events is not known but anticoagulation is often empirically prescribed. Recommendations on antithrombotic treatment and PCI for acute coronary syndromes should be maintained during COVID-19 treatment

ARDS: acute respiratory distress syndrome; PCI: percutaneous coronary intervention (adapted from Nishiga et al.^11^ and Prieto-Lobato et al.^12^)

Concerning coronary artery disease epidemiology, some studies showed a decreased incidence of hospitalization for acute myocardial infarction during the COVID-19 pandemic. Solomon et al.^13^ noted that the weekly rates of hospitalization for acute myocardial infarction decreased by up to 48% during the COVID-19 period. De Filippo et al.^14^ found a similar decrease in hospitalization for acute coronary syndrome in 15 hospitals in northern Italy.^14^ This decrease is partially related to the anxiety and fear of catching COVID-19 in the emergency department that many patients demonstrated during the initial months of the pandemic. Due to this decrease in myocardial infarction hospitalizations, a transient increase in out-of-hospital cardiac arrest (AOHCA) was observed, compared to the equivalent time period in the years before the pandemic.^15^ This increase of AOHCA is directly attributable to COVID-19 infections and to the potential increase of patients with acute coronary syndrome that did not go immediately to emergency settings.

We must congratulate the authors of the article published in Arquivos Brasileiros de Cardiologia.^1^ Their main findings show a gradual increase in the rate of out-of-hospital cardiac arrest during the coronavirus disease 2019 (COVID-19) pandemic in the city of Belo Horizonte, Minas Gerais, Brazil. Their data demonstrates a proportional increase of 33% of home deaths in March 2020 compared to previous periods. Their study is the first Brazilian paper to demonstrate the same trend observed in other countries.^14,15^ The main limitations of the study are: short observation period, sample from a single Brazilian metropolitan region and lack of complete information on comorbidities in about 40% of cases. However, these data do not invalidate the main messages of the study, which are: (1) the need to organize the health system to deal with cases of acute diseases during the COVID-19 pandemic, (2) educating the population about the need to seek continued health care, and (3) the search for better treatments and prevention.

The approval of effective vaccines for the prevention of COVID-19 and the beginning of the Brazilian national immunization program against COVID-19 in January 2021 fill us with hope and optimism.^16,17^ However, as long as vaccination is not widely available to the population, it is necessary to continue with effective and scientifically proven measures of social distancing, the use of masks and hand hygiene. Only then will the COVID-19 pandemic be a page in history and no longer a harsh reality.
